# Biomarkers in Neuropsychiatric Systemic Lupus Erythematosus: A Systematic Literature Review of the Last Decade

**DOI:** 10.3390/brainsci12020192

**Published:** 2022-01-30

**Authors:** Julius Lindblom, Chandra Mohan, Ioannis Parodis

**Affiliations:** 1 Division of Rheumatology, Department of Medicine Solna, Karolinska Institute and Karolinska University Hospital, 17176 Stockholm, Sweden; julius.lindblom@ki.se; 2 Department Biomedical Engineering, University of Houston, Houston, TX 77204, USA; cmohan@central.uh.edu; 3 Department of Rheumatology, Faculty of Medicine and Health, Örebro University, 70182 Örebro, Sweden

**Keywords:** systemic lupus erythematosus, neuropsychiatric systemic lupus erythematosus, biomarkers, diagnosis, monitoring, prognosis

## Abstract

Nervous system involvement in patients with SLE, termed neuropsychiatric SLE (NPSLE), constitutes a diagnostic challenge, and its management is still poorly optimised. This review summarises recent insights over the past decade in laboratory biomarkers of diagnosis, monitoring, and prognosis of NPSLE. An initial systematic search in the Medline and Web of Science was conducted to guide the selection of articles. Emerging diagnostic biomarkers in NPSLE that displayed satisfactory ability to discriminate between NPSLE and controls include serum interleukin (IL)-6, microRNA (miR)-23a, miR-155, and cerebrospinal fluid (CSF) α-Klotho. CSF lipocalin-2, macrophage colony-stimulating factor (M-CSF), and immunoglobulin (Ig)M also displayed such ability in two ethnically diverse cohorts. Serum interferon (IFN)-α and neuron specific enolase (NSE) were recently reported to moderately correlate with disease activity in patients with active NPSLE. CSF IL-8, IL-13, and granulocyte colony-stimulating factor (G-CSF) exhibited excellent sensitivity, yet poorer specificity, as predictors of response to therapy in patients with NPSLE. The overall lack of validation studies across multiple and diverse cohorts necessitates further and well-concerted investigations. Nevertheless, we propound CSF lipocalin 2 among molecules that hold promise as reliable diagnostic biomarkers in NPSLE.

## 1. Introduction

Involvement of the central and peripheral nervous systems is a common but poorly understood manifestation of systemic lupus erythematosus (SLE), termed neuropsychiatric SLE (NPSLE). Although studies have reported varying prevalence estimates [1], largely depending on the level of stringency in definitions, NPSLE affects at least 20% of patients with SLE within the first years of the disease course [2]. Similar to other organ manifestations in SLE, NPSLE is highly heterogeneous and is commonly stratified into 19 different neuropsychiatric syndromes according to the 1999 American College of Rheumatology (ACR) NPSLE classification, including 12 central nervous system (CNS) and 7 peripheral nervous system (PNS) manifestations [3]. The CNS manifestations can be further subdivided into focal neurological deficits and diffuse psychiatric or neuropsychological syndromes, as shown in Table 1 [3]. It remains a challenge to distinguish these diverse clinical features from neuropsychiatric events unrelated to SLE. The state of the art in diagnostics relies on multidisciplinary approaches and expert-based attribution of the neuropsychiatric symptoms to SLE upon exclusion of other causes, following an extensive diagnostic workup that includes laboratory assessment and neuroimaging [4]. The management of NPSLE is poorly optimised due to the lack of high-level evidence in the literature and comprises, to a large extent, symptomatic strategies rather than specific treatment of the underlying causes [5,6,7,8]. The purpose of this review was to summarise recent insights in laboratory biomarkers of diagnosis, monitoring, and prognosis of NPSLE, based on literature over the past decade. Among the biomarkers reviewed, CSF lipocalin 2 holds promise as a reliable diagnostic biomarker in NPSLE. Overall, NPSLE appears scarcely researched, and the vast majority of biomarker investigations lack validation across multiple and diverse cohorts.

## 2. Methods

An initial systematic search for relevant articles was performed as described in the online Supplementary Material (Figure S1; Tables S1 and S2), including a Preferred Reporting Items for Systematic reviews and Meta-Analyses (PRISMA) 2020 flow diagram (Figure S1) [9]. In short, a search of the Medline and Web of Science for laboratory biomarker studies in English language comprising adult patients with NPSLE published between 1 January 2012 and 14 January 2022 was conducted. The search strategy and terms are detailed in the online Supplementary Material (Tables S1 and S2). Animal studies were beyond the scope of this review. The risk of bias (RoB) in the included studies was assessed using the Joanna Briggs Institute (JBI) Critical Appraisal Checklist for Analytical Cross-Sectional Studies, the JBI Critical Appraisal Checklist for Systematic Reviews and Research Syntheses, and the JBI Critical Appraisal Checklist for Cohort Studies [10], as appropriate.

### 2.1. Diagnostic Biomarkers

Recent literature reporting diagnostic biomarkers in NPSLE is summarised in Table 2. Serum anti-β_2_-glycoprotein I (β_2_GPI) antibodies of immunoglobulin (Ig)G and/or IgM isotypes displayed an odds ratio (OR) of 2.5 (95% confidence interval [CI]: 1.2–5.2) for distinguishing NPSLE from non-neuropsychiatric SLE in a large Italian cohort of SLE patients [11]. However, earlier studies failed to show such an association [12,13,14]. Nevertheless, in a study of focal NPSLE by Hawro et al. [15], serum anti-β_2_GPI IgM yielded an OR of 5.6 (95% CI: 1.2–26.9) for ischemic stroke and anti-β_2_GPI IgG yielded an OR of 11.3 (95% CI: 2.0–63.0) for seizures in a smaller cohort of SLE patients. A similar albeit weaker (OR: 6.5; 95% CI: 1.3–31.9) association was found for serum anti-cardiolipin (aCL) IgA and seizures [15]. These results are in line with previous reports of a link between anti-phospholipid (aPL) antibodies and neuropsychiatric manifestations of SLE, including cerebrovascular disease and seizures, supporting the role of vascular pathogenetic mechanisms in a substantial fraction of NPSLE patients [16,17,18,19]. Furthermore, the association between serum anti-ribosomal P antibodies and NPSLE (OR: 2.0; 95% CI: 1.2–3.4) was recently corroborated [20,21] and reported to be even stronger for diffuse involvement e.g., psychosis (OR: 3.1; 95% CI: 1.9–4.9) in a meta-analysis by Choi et al. [20]. Interestingly, anti-ribosomal P antibodies purified from patients with NPSLE were shown to induce apoptosis in hippocampal neurons and impair memory in mice [22].

Emerging diagnostic protein and cytokine biomarkers in NPSLE that have displayed satisfactory ability to discriminate between NPSLE and non-neuropsychiatric SLE include serum interleukin (IL)-6 (area under the curve [AUC]: 0.89; *p*-value not available) [23], high-mobility group box protein 1 (HMGB1; AUC: 0.84; *p* < 0.05) [24] and cerebrospinal fluid (CSF) α-Klotho (AUC: 0.94; *p* < 0.001) [25], as detailed in Table 2. IL-6 serves an important function in stimulating activated B cells to secrete Ig and is known to be elevated in the CSF of NPSLE patients [26,27]. HMGB1 is a DNA-binding protein exerting various effects on immune cells but also on neuronal cells [28]. As HMGB1 has been reported as a marker of SLE disease activity [29] and an HMGB1 antagonist was shown to decrease seizure recurrence in mice [30], HMGB1 may constitute a target for future drug development in NPSLE. Interestingly, the single-pass transmembrane protein α-Klotho has been reported to have anti-inflammatory properties as well as regulate age-related cognitive decline, and reduced α-Klotho levels have been associated with myelin degradation [31,32,33]. CSF lipocalin-2, macrophage colony-stimulating factor (M-CSF), and IgM displayed good ability to distinguish patients with NPSLE from healthy individuals and/or patients with other neurological diseases in two separate cohorts of Canadian and Chinese patients (AUC: 0.80–0.85; *p* < 0.001 for all, AUC: 0.71–0.91; *p* < 0.05 for all, and AUC: 0.78–0.95; *p* < 0.01 for all, respectively) [34,35,36]. Lipocalin 2 is an acute-phase glycoprotein that is secreted by neurons among multiple cell types during cellular stress [37,38]. M-CSF expressing T helper (T_h_) cells were recently reported to be elevated in CSF from patients with MS, a neurological disease that resembles NPSLE in terms of certain neuroinflammatory attributes, while CSF IgG index was associated with inflammatory activity in magnetic resonance imaging (MRI) of the brain in MS patients [39,40].

Using a novel proteomic approach to investigate immune complex (IC)-associated antigens in CSF in patients with NPSLE, Aibara et al. [41] discovered several proteins with excellent specificity, yet overall poor sensitivity for distinguishing NPSLE patients from healthy controls (Table 2). Among those, occurrence of CSF suprabasin isoform 1 precursor displayed the best diagnostic properties, with a specificity of 100% but a sensitivity of 35% [41]. These results prompted further investigation by Ichinose et al. [42], who reported that CSF anti-suprabasin antibodies could distinguish patients with NPSLE from patients with non-neuropsychiatric SLE, multiple sclerosis (MS), and normal pressure hydrocephalus (NPH) in a Japanese cohort (N = 103), with a specificity of 92% and a sensitivity of 42% using an antibody index (A.I.) cut-off of 1.0. Results from the same study lent support for a pathogenetic role of anti-suprabasin antibodies through alterations of senescence and autophagy pathways in astrocytes [42].

A common single nucleotide polymorphism (SNP) i.e., rs11797 of the three prime repair exonuclease 1 (TREX1) gene was reported to be more frequent in patients with NPSLE than in SLE patients without any non-neuropsychiatric manifestations with an OR of 6.4 (95% CI: 1.7–26.2) [43]. Similarly, an earlier study found rs11797 to discriminate between SLE patients with focal involvement i.e., seizures and healthy controls (OR: 1.7; 95% CI: 1.2–2.4) [44]. Mutations in the TREX1 gene have been linked to several diseases, including SLE and Aicardi-Goutières syndrome (AGS), a rare neurological condition that is characterized by its onset in early childhood and bears some clinical resemblance with SLE [45]. Emerging epigenetic biomarkers in NPSLE that have displayed satisfactory ability to discriminate between NPSLE and controls (healthy individuals or other neurological diseases) include microRNA (miR)-23a (AUC: 0.95–0.98; *p* < 0.001 for all) [46] and miR-155 (AUC: 0.76–0.92; *p* < 0.05 for all) [46]. MicroRNAs (miRNAs) are short single-stranded RNA molecules that regulate gene expression through degradation of messenger RNAs [47] and have been indicated to have a role in disease mechanisms of SLE in recent epigenetic research [48,49].

**Table 2:** Performance of selected diagnostic biomarkers in NPSLE.

Biomarker	Sample	Feature	Comparator	Metrics	References
Antibodies					
Anti-ribosomal P (+)	Serum/ plasma	Unspecified	Non-NP SLE	AUC: 0.57; OR: 2.0–3.3	Huang et al., 2020 [24]; Zhang et al., 2021 [21]
		Unspecified; Diffuse		Pooled OR: 1.6–3.1	Choi et al., 2020 [20]
Anti-Sm (+)	Serum/ plasma	Focal; Unspecified	Non-seizure SLE; Non-NP SLE, MS, NMO and VM	OR: 1.0–3.3	Mikdashi et al., 2005 [17]; Ushigusa et al., 2016 [25]
		Unspecified	Non-NP SLE, MS, NMO and VM	Adj. OR: 0.9	Ushigusa et al., 2016 [25]
aCLs * (+)	Serum/ plasma	Unspecified; Focal	Non-NP SLE; Non-seizure SLE	OR: 1.9–7.3	Govoni et al., 2012 [11]; Karassa et al., 2000 [18]; Hawro et al., 2015 [15]; Mikdashi et al., 2005 [17]
		Unspecified	Non-NP SLE	Adj. OR: 3.1	Mok et al., 2001 [16]
		Focal	Non-seizure SLE	HR: 2.2	Mikdashi et al., 2005 [17]
Anti-β_2_GP1 * (+)	Serum/ plasma	Unspecified; Focal	Non-NP SLE; Non-CVD SLE; Non-headache SLE; Non-seizure SLE	OR: 2.5–11.3	Govoni et al., 2012 [11]; Hawro et al., 2015 [15]
Anti-GAPDH (levels)	Serum/ plasma	Unspecified	N/A	ρ = 0.57	Sun et al., 2019 [50]
Anti-GABAR ^†^ (+)	Serum/ plasma	Unspecified	Non-NP SLE	OR: 4.6–5.5	Tsuchiya et al., 2014 [51]
Anti-vimentin ^‡^ (<40.5 NFI)	Serum/ plasma	Unspecified	Non-NP SLE	Sens.: 88%; Spec.: 66%; AUC: 0.81; OR: 13.5	van der Meulen et al., 2017 [52]
Anti-heparan sulphate ^‡^ (>20.5 NFI)				Sens.: 65%; Spec.: 70%; AUC: 0.72; OR: 4.5	
Anti-nucleoporin 62 ^‡^ (<26.5 NFI)				Sens.: 81%; Spec.: 55%; AUC: 0.72; OR: 4.5	
Anti-prothrombin ^‡^ (<32.5 NFI)				Sens.: 65%; Spec.: 65%; AUC: 0.69; OR: 3.6	
Anti-glycoprotein 2 ^‡^ (<34.5 NFI)				Sens.: 68%; Spec.: 65%; AUC: 0.68; OR: 4.0	
Anti-cardiolipin ^‡^ (>0.5 NFI)				Sens.: 45%; Spec.: 87%; AUC: 0.66; OR: 5.3	
Anti-histone H2A ^‡^ (>189.0 NFI)				Sens.: 61%; Spec.: 64%; AUC: 0.65; OR: 2.7	
Anti-histone H2B ^‡^ (>146.5 NFI)				Sens.: 64%; Spec.: 63%; AUC: 0.65; OR: 2.9	
Anti-collagen II ^‡^ (>4.5 NFI)				Sens.: 65%; Spec.: 58%; AUC: 0.65; OR: 2.6	
Anti-heparin ^‡^ (>174.0 NFI)				Sens.: 65%; Spec.: 61%; AUC: 0.65; OR: 2.8	
Anti-amyloid ^‡^ (>1.5 NFI)				Sens.: 70%; Spec.: 58%; AUC: 0.65; OR: 3.2	
Anti-suprabasin (A.I. ≥1.0)	CSF	Unspecified	Non-NP SLE, MS and NPH	Sens.: 42%; Spec.: 92%; AUC: 0.78	Ichinose et al., 2018 [42]
Proteins/cytokines					
C3 (low)	Serum/ plasma	Unspecified	Non-NP SLE	OR: 3.8	Karassa et al., 2000 [18]
C3 (levels)			Non-NP SLE, MS, NMO and VM	Adj. OR: 1.1	Ushigusa et al., 2016 [25]
NfL (↑)	Serum/ plasma	Unspecified	Non-NP SLE	AUC: 0.65	Engel et al., 2021 [53]
NSE (levels)	Serum/ plasma	Unspecified	Non-NP SLE	ρ = −0.37	Hawro et al., 2015 [54]
HMGB1 (levels)	Serum/ plasma	Unspecified	Non-NP SLE	AUC: 0.84; OR: 1.7	Huang et al., 2020 [24]
IL-6 (>74.9 pg/mL; ↑)	Serum/ plasma	Unspecified	Non-NP SLE	Sens.: 75%; Spec.: 100%; AUC: 0.89	Kitagori et al., 2019 [23]
	CSF		MS and NMO	Sens.: 22%; Spec.: 93%; PPV: 70%; NPV: 61%	Ichinose et al., 2015 [55]
ApoA1 (levels)	Serum/ plasma	Diffuse	N/A	ρ = 0.21	Lu et al., 2021 [56]
ApoE (levels)				ρ = −0.21	
Free T3 (levels)				ρ = 0.19–0.32	
Free T4 (levels)				ρ = 0.28–0.42	
HDL-C (levels)				ρ = 0.05–0.08	
IGFBP7 (levels)				ρ = −0.22	
S100B (>0.0218 ng/mL)	Serum/ plasma	Unspecified	Non-NP SLE	Sens.: 81%; Spec.: 67%; PPV: 84%; NPV: 62%; AUC: 0.74	Noris-García et al., 2018 [57]
IL-17 (↑)	CSF	Unspecified	MS and NMO	Sens.: 91%; Spec.: 90%; PPV: 88; NPV: 93	Ichinose et al., 2015 [55]
IL-2 (↑)				Sens.: 88%; Spec.: 93%; PPV: 90; NPV: 91	
IFN-γ (↑)				Sens.: 88%; Spec.: 98%; PPV: 97; NPV: 91	
IL-5 (↑)				Sens.: 88%; Spec.: 98%; PPV: 97; NPV: 91	
FGF2 (↑)				Sens.: 88%; Spec.: 95%; PPV: 93; NPV: 91	
IL-15 (↑)				Sens.: 94%; Spec.: 95%; PPV: 94; NPV: 95	
IL-8 (↑)				Sens.: 22%; Spec.: 93%; PPV: 70; NPV: 61	
Osteopontin (>963.4 ng/mL)	CSF	Unspecified	Non-NP SLE	Sens.: 70%; Spec.: 100%; AUC: 0.88	Kitagori et al., 2019 [23]
Lipocalin 2 (↑, ≥122 pg/mL; ≥126 pg/mL;)	CSF	Unspecified	HC/other neurological diseases	Sens.: 76–94%; Spec.: 80%; PPV: 63–84%; NPV: 88–92%; AUC: 0.80–0.85	Mike et al., 2019 [35]; Vanarsa et al., 2022 (in print) [34]
α-Klotho (≤230.2 pg/mL)	CSF	Unspecified	Non-NP SLE, MS, NMO and VM	Sens.: 82%; Spec.: 94%; AUC: 0.94; OR: 0.98	Ushigusa et al., 2016 [25]
Angiostatin (≥12 ng/mL)	CSF	Unspecified	HC/other neurological diseases	Sens.: 88%; Spec.: 44%; PPV: 45%; NPV: 88%; AUC: 0.65	Vanarsa et al., 2022 (in print) [34]
DAN (≥21,457 pg/mL)				Sens.: 76%; Spec.: 63%; PPV: 52%; NPV: 84%; AUC: 0.75	
Fibronectin (≥3539 pg/mL)				Sens.: 67%; Spec.: 85%; PPV: 70%; NPV: 83%; AUC: 0.81	
HCC-1 (≥3665 pg/mL)				Sens.: 52%; Spec.: 85%; PPV: 65%; NPV: 78%; AUC: 0.69	
M-CSF (≥41 pg/mL, ≥95 pg/mL)				Sens.: 47–80%; Spec.: 94–100%; PPV: 87–100%; NPV: 62–90%; AUC: 0.71–0.91	
SERPING1 (≥415 ng/mL)				Sens.: 71%; Spec.: 80%; PPV: 65%; NPV: 85%; AUC: 0.78	
IgM (≥1220 ng/mL, ≥5586 ng/mL)				Sens.: 70–100%; Spec.: 89–100%; PPV: 83–100%; NPV: 75–100%; AUC: 0.78–0.95	
IC-associated antigens					
Isoform 7 of nesprin-1 (+)	CSF (IC-associated)	Unspecified	HC	Sens.: 8%; Spec.: 100%	Aibara et al., 2018 [41]
Suprabasin isoform 1 precursor (+)				Sens.: 35%; Spec.: 100%	
Calmodulin-like protein 5 (+)				Sens.: 12%; Spec.: 92%	
cDNA FLJ58075, highly similar to ceruloplasmin (+)				Sens.: 4%; Spec.: 96%	
Desmoglein-1 (+)				Sens.: 15%; Spec.: 96%	
INTS4-like protein 2 (+)				Sens.: 4%; Spec.: 96%	
Isoform 1 of α1-antitrypsin (+)				Sens.: 8%; Spec.: 98%	
Isoform 2 of NUMA1 (+)				Sens.: 4%; Spec.: 96%	
Protein piccolo (+)				Sens.: 8%; Spec.: 98%	
Isoform 3 ofRICTOR (+)				Sens.: 19%; Spec.: 100%	
Genetic/epigenetic markers					
PD-1 (FC)	Blood (gene expression)	Diffuse	Non-psychosis SLE	ρ = 0.24	Bassiouni et al., 2021 [58]
TREX1 (relative frequencies)	Blood (SNPs ^§^)	Unspecified; Focal	Non-NP SLE; HC; Non-neurological SLE	OR: 1.6–44.7	Fredi et al., 2015 [43]; Namjou et al., 2011 [44]
TRPC6 (relative frequencies)	Blood (SNP rs7925662)	Unspecified	Non-NP SLE	HR: 0.4–3.3	Ramirez et al., 2015 [59]; Ramirez et al., 2018 [60]
miR-145 (FC > 0.0041; FC > 0.92; FC > 0.61)	Serum/ plasma (expression)	Unspecified	HC; MS; NMO	Sens.: 60–80%; Spec.: 83–100%; PPV: 57–100%; NPV: 77–94%; AUC: 0.76–0.90	Sharaf-Eldin et al., 2017 [61]
miR-223 (FC > 0.925; FC > 0.325; FC > 0.61)				Sens.: 90%; Spec.: 87–100%; PPV: 75–100%; NPV: 90–97%; AUC: 0.91–0.99	
miR-326 (FC > 0.0037; FC > 0.505; FC > 0.61)				Sens.: 90%; Spec.: 78–90%; PPV: 64–90%; NPV: 90–97%; AUC: 0.82–0.90	
SMAD3 (FC > 0.052)	Serum/ plasma (gene expression)	Unspecified	HC	Sens.: 70%; Spec.: 78%; PPV: 58%; NPV: 86%; AUC: 0.73	Sharaf-Eldin et al., 2017 [61]
SP1 (FC > 0.795)			MS	Sens.: 80%; Spec.: 76%; PPV: 47%; NPV: 93%; AUC: 0.82	
miR-23a (FC ≥ 0.1; FC ≥ 7.3)	Serum/ plasma (expression)	Unspecified	HC; MS	Sens.: 90–100%; Spec.: 96–100%; AUC: 0.95–0.98	Sharaf-Eldin et al., 2020 [46]
miR-155 (FC ≥ 0.1; FC ≥ 7.3)			HC; NMO; MS	Sens.: 60–90%; Spec.: 88–90%; AUC: 0.76–0.92	
miR-572 (FC ≥ 4.5)			HC	Sens.: 90%; Spec.: 68%; AUC: 0.80	

aCLs: anti-cardiolipins; adj.: adjusted; A.I.: antibody index; ApoA1: apolipoprotein A1; ApoE: apolipoprotein E; AUC: area under the curve; β_2_GPI: β_2_-glycoprotein 1; C3: complement component 3; cDNA; circular deoxyribonucleic acid; CSF: cerebrospinal fluid; CVD: cerebrovascular disease; FC: fold change; FGF2: fibroblast growth factor 2; GABAR: gamma-aminobutyric acid type B receptors; GAPDH: glyceraldehyde 3-phosphate dehydrogenase; HC: healthy control; HDL-C: high-density lipoprotein cholesterol; HMGB1: high-mobility group box protein 1; HR: hazard ratio; IC: immune complex; IFN: interferon; Ig: immunoglobulin; IGFBP7: insulin-like growth factor binding protein 7; IL: interleukin; INTS4: integrator complex subunit 4; M-CSF: macrophage colony-stimulating factor; miR: microRNA; NFI: net fluorescent intensity; NPH: normal pressure hydrocephalus; NP: neuropsychiatric; NPSLE: neuropsychiatric systemic lupus erythematosus; NSE: neuron specific enolase; MS: multiple sclerosis; NMO: neuromyelitis optica; NPV: negative predictive value; NUMA1: nuclear mitotic apparatus protein 1; OR: odds ratio; PD: programmed death 1; PPV: positive predictive value; RICTOR: rapamycin-insensitive companion of mammalian target of rapamycin; S100B: S100 calcium-binding protein B; Sens.: sensitivity; SERPING1: serpin family G member 1; SLE: systemic lupus erythematosus; Sm: Smith; SMAD3: signaling mother against decapentaplegic peptide 3; SNP: single nucleotide polymorphism; SP1: specificity protein 1; Spec.: specificity; T3: triiodothyronine; T4: thyroxine; TREX1: three prime repair exonuclease 1; TRPC6: transient receptor potential cation channel, subfamily C, member 6; VM: venous malformation; ↑: elevated. * IgA, IgG and/or IgM isotypes; ^†^ anti-GABAR_B1b_ and anti-GABAR_B2_ antibodies; ^‡^ IgG isotype; ^§^ rs11797, rs922075, rs6776700, rs6442123, rs2242150, or rs3135945.

### 2.2. Biomarkers of Disease Activity

Serum interferon (IFN)-α exhibited a trend towards a moderate correlation (ρ = 0.33; *p* = 0.05) with disease activity measured with the Systemic Lupus Erythematosus Disease Activity Index 2000 (SLEDAI-2K) [62] in patients with NPSLE at the onset of neuropsychiatric manifestations [63]. Type I IFNs are considered key mediators in SLE pathogenesis [64], with the recent approval of the anti-type I IFN receptor monoclonal anifrolumab [65,66] by the US Food and Drug Administration (FDA) for the treatment of SLE [67], lending cogent credence to this notion. By contrast, serum neuron-specific enolase (NSE) displayed a negative correlation with disease activity (ρ = −0.42; *p* < 0.05) measured with the Systemic Lupus Activity Measure (SLAM) [68] in patients with active NPSLE [54]. NSE is a glycolytic enzyme that is expressed in neural cells [69]. Peripheral blood levels of NSE have been shown to increase upon acute neuronal injury, whereas reduced levels have been reported in chronic diseases of the CNS, and a negative correlation between plasma NSE and disease progression was reported in patients with MS [70,71,72,73].

Fatigue is a major complaint in patients with SLE and has been linked to SLE disease activity [74], as well as cognitive impairment in NPSLE [75]. A study by Hopia et al. [76] comprising 28 patients with NPSLE from a Swedish clinical setting showed a positive correlation (ρ = 0.43; *p* < 0.05) between CSF levels of a proliferation-inducing ligand (APRIL) and fatigue measured with the Fatigue Severity Scale (FSS) [77].

### 2.3. Biomarkers of Response to Therapy

Ichinose et al. [78] recently investigated the performance of selected molecules measured in CSF from patients with NPSLE (N = 28) as predictors of response to conventional immunosuppressive therapy with or without the addition of rituximab, evaluated 1 year after treatment commencement. Several markers displayed excellent sensitivity yet poorer specificity for predicting treatment response, with downregulated IL-8 (sensitivity: 100%; specificity: 50%), IL-13 (sensitivity: 100%; specificity: 50%), and G-CSF (sensitivity: 100%; specificity: 50%) displaying the best overall metrics [78].

### 2.4. Prognostic Biomarkers

Data on prognostic biomarkers of long-term outcome in NPSLE were scarce. In a study from the large Hopkins Lupus Cohort by Huang et al. [79] of which 46% of patients were followed up for more than 5 years, a history of anti-Smith (Sm) antibodies (rate ratio [RR]: 1.8; *p* = 0.005), low complement component 3 (C3; RR: 2.0; *p* = 0.001), low C4 (RR: 1.7; *p* = 0.006), and urine dipstick protein 3+ at the most recent visit (RR: 7.4; *p* < 0.001) predicted incidence of seizures upon SLE diagnosis, after adjustment for age. However, only a history of low C3 (RR: 1.8, *p* = 0.008) and urine dipstick protein 3+ (RR: 2.7; *p* = 0.004) remained significant in multivariable analyses (79). A study by Zhang et al. (21) showed that elevated serum creatinine levels were an independent contributor to premature mortality in patients with NPSLE with an adjusted hazard ratio (HR) of 3.3 (95% CI: 1.1–9.5) in multivariable regression analysis, which however is a finding with no apparent manifestation-specific relevance.

## 3. Perspective

Although in-depth understanding of the pathogenesis of NPSLE remains poor, technological advances are anticipated to help shed light on this complex SLE manifestation, and investigations of the last decade have contributed useful insights for a more appropriate management of these patients. However, while research has focused on optimisation of diagnostic procedures through the derivation of novel biomarkers, little has been done with regard to surveillance and prognosis of long-term outcomes. Moreover, while several candidate markers have emerged, the study populations have overall been limited and heterogenous, partly owing to the rarity of the different NPSLE syndromes. Most of the studies included in the present review investigated associations with the entire NPSLE spectrum i.e., all items in the 1999 ACR classification [3] together rather than as distinct groups of NPSLE patients e.g., patients with focal neurological or diffuse psychiatric or neuropsychological manifestations. In this respect, it is worth noting that studies that focused on either focal or diffuse manifestations investigated serum or plasma biomarkers. Since CSF constitutes a source of sampling that is more specific to the CNS, future CSF biomarker survey might be useful towards identification of manifestation-specific mediators and acquirement of further insights into underlying pathogenetic mechanisms. Among emerging diagnostic biomarkers, CSF α-Klotho and serum IL-6, miR-23a and miR-155 exhibited satisfactory ability to discriminate between patients with NPSLE and controls, as did CSF lipocalin-2, M-CSF, and IgM in two ethnically diverse cohorts. Serum IFN-α and NSE were reported to moderately correlate with disease activity in patients with NPSLE, while CSF IL-8, IL-13, and G-CSF exhibited excellent sensitivity, yet poorer specificity, as predictors of response to therapy.

In our assessment of RoB, sources of bias primarily included three bias types i.e., some studies did not describe the subjects and the setting in detail, some did not use standard classification to define NPSLE, and some did not account for confounding factors, as indicated in the online Supplementary Material (Tables S3–S5). The overall lack of validation studies across multiple cohorts necessitates further and well-concerted investigations.

In contrast with more clearly defined neurological conditions e.g., MS, where imaging constitutes an invaluable guide of diagnosis and monitoring, NPSLE still suffers challenges with regard to definite classification, owing to its immense heterogeneity and, partly as a consequence of the latter, inconsistent nomenclature in the literature. Thus, we advocate bolstering the classification of distinct syndromes within the clinical spectrum of NPSLE, with validated candidate biomarkers emerging from detailed molecular characterisation of these distinct clinical phenotypes. We foresee that broad molecular and cellular profiling through next-generation sequencing, cutting-edge proteomic and metabolomic investigations, and data integration with sophisticated bioinformatics that incorporate systems biology will revolutionise the field of NPSLE and pave the way toward person-centred diagnostics, and management and prevention of long-term damage, flares, or even disease occurrence. A prerequisite for research with such premise would be broad multicentre collaborations beyond country or continental borders.

## Figures and Tables

**Table 1:** Neuropsychiatric syndromes according to the 1999 ACR NPSLE classification [3].

CNS		PNS
Diffuse	Focal	
Acute confusional state	Aseptic meningitis	Acute inflammatory demyelinating polyradiculoneuropathy
Anxiety disorders	Cerebrovascular disease	Autonomic disorder
Cognitive dysfunction	Demyelinating syndromes	Mononeuropathy (single/multiplex)
Mood disorders	Headache	Myasthenia gravis
Psychosis	Movement disorder	Cranial neuropathy
	Myelopathy	Plexopathy
	Seizure disorders	Polyneuropathy

Adapted from “The American College of Rheumatology nomenclature and case definitions for neuropsychiatric lupus syndromes.” Arthritis Rheum. 1999;42(4):599–608. Copyright 1999 by the American College of Rheumatology. Adapted with permission. ACR: American College of Rheumatology; CNS: central nervous system; PNS: peripheral nervous system; NPSLE: neuropsychiatric systemic lupus erythematosus.

## Supplementary Materials

The following are available online at https://www.mdpi.com/article/10.3390/brainsci12020192/s1, Figure S1: PRISMA 2020 flow diagram for new systematic reviews that included searches of databases and registers only; Table S1. Search in the Medline; Table S2. Search in the Web of Science; Table S3. Risk of bias assessment of cross-sectional studies; Table S4. Risk of bias assessment of meta-analyses; Table S5. Risk of bias assessment of cohort studies.
