# Supplementary material for: Biomarkers in Neuropsychiatric Systemic Lupus Erythematosus: A Systematic Literature Review of the Last Decade
Source: Brain Sci. 2022 Jan 30;12(2):192. doi: 10.3390/brainsci12020192 (PMC8869794; doi:10.3390/brainsci12020192)
Supplement: Supplementary file 1 [file brainsci-12-00192-s001.zip › Table S1.pdf]

Table S1. Search in the Medline

Interface: Ovid MEDLINE(R) and Epub Ahead of Print, In-Process & Other Non-Indexed Citations and Daily<br><br>Date of Search: 7 December 2021<br><br>Number of hits: 1799<br><br>Comment: In Ovid, two or more words are automatically searched as phrases; i.e. no quotation marks are needed	Field labels<br><br>• exp/ = exploded MeSH term<br>• / = non exploded MeSH term<br>• .ti,ab,kf. = title, abstract and author keywords<br>• adjx = within x words, regardless of order<br>• * = truncation of word for alternate endings

#	Query	Hits	Comments
**Block 1: SLE**			
1	exp Lupus Vasculitis, Central Nervous System/	903	
2	lupus.jw.	5,982	Journal dedicated to SLE.
3	(lupus adj3 (auto-immun* or autoimmun* or disseminat* or erythemat* or nephriti* or neuropsych* or neuro-psych* or systemic*)).ti,ab,kf.	66,944	Terms choosen from MeSH (incl entry terms).
4	(SLE and lupus).ti,ab,kf.	32,477	Both SLE and lupus can be interpreted otherwise. Thus, combination was chosen.
5	or/1-4	69,505	
**Block 2: Biomarkers**			
6	exp Biomarkers/	818,536	Includes the following narrower terms: “Antibodies, Antineutrophil Cytoplasmic”, “Antigens, Differentiation”, “Biomarkers, Pharmacological”, “Biomarkers, Tumor”, “Chorionic Gonadotropin, beta Subunit, Human”, “Environmental Biomarkers”, “Fibrinopeptide A”, “Genetic Markers”, “Oligoclonal Bands”
7	(biomarker* or marker*).jw.	20,588	
8	(biomarker* or biological marker* or diagnostic marker*).ti,ab,kf.	352,017	Biological and diagnostic causes too much noise with ADJx.
9	((immunol* or metabol* or molecular* or novel) adj3 marker*).ti,ab,kf.	57,773	ADJ3 reasonable, since more than two words in between gives much noise.
10	((blood or cerebrospinal or plasma or sera or serologic* or serum or urinary or urine) adj3 marker*).ti,ab,kf.	39,363	Terms for where markers may be found. See #9.
11	((antibod* or antigen* or autoantibod* or cytokine* or glycoprotein* or immunoglob* or lectin* or lipoprotein* or peptide* or protein* or RNA) adj3 marker*).ti,ab,kf.	34,682	Terms for substances commonly occurring as biomarkers. See #9.
12	or/6-11	1,100,776	
13	exp Peptides/	2,863,960	MeSH-terms for substances occurring as biomarkers, to be
14	exp Blood Proteins/	1,684,860	

15	exp Membrane Proteins/	2,006,797	combined with free-text word marker*.<br><br>Terms included if giving > 10 hits in combination with block 1 and MeSH-term Biomarkers/.
16	exp Carrier Proteins/	1,230,856	
17	exp Glycoproteins/	877,057	
18	exp Lipoproteins/	153,035	
19	exp DNA-Binding Proteins/	590,421	
20	exp Cytoskeletal Proteins/	297,813	
21	exp "Receptors, Cytoplasmic and Nuclear"/	169,181	
22	exp Lectins/	115,752	
23	or/13-22	5,985,307	
24	marker*.ti,ab,kf.	838,081	
25	23 AND 24	352,958	
26	12 or 25	1,272,019	
**Block 3: Study types for exclusion**			
27	exp Animals/ not Humans/	4,924,285	Based on a filter from McGill University.
28	exp Models, Animal/ not Humans/	422,080	
29	exp Animal Experimentation/ not Humans/	5,691	
30	(animal or animals or canine or dog or dogs or feline or hamster or hamsters or lamb or lambs or mice or monkey or monkeys or mouse or murine or pig or pigs or piglet* or porcine or primate or primates or rabbit or rabbits or rat or rats or rodent* or sheep or sheeps or veterinar*).ti,kf,jw.	2,544,152	
31	or/27-30	5,431,043	
32	case reports.pt.	2,230,033	
33	case.jw.	113,552	
34	(case adj (report* or series or stud*)).ti,kf.	391,780	ADJ1 (or more) include case-control studies.
35	or/33-34	477,444	
36	31 OR 35	5,902,092	
**Result: Biomarkers in SLE**			
37	(5 and 26) not 36	6,820	
38	limit 37 to (yr="2012 -Current" and "all adult (19 plus years)" and english)	1,800	Limits based on exclusion criteria.
