# Supplementary material for: Biomarkers in Neuropsychiatric Systemic Lupus Erythematosus: A Systematic Literature Review of the Last Decade
Source: Brain Sci. 2022 Jan 30;12(2):192. doi: 10.3390/brainsci12020192 (PMC8869794; doi:10.3390/brainsci12020192)
Supplement: Supplementary file 1 [file brainsci-12-00192-s001.zip › Table S2.pdf]

Table S2. Search in the Web of Science

Interface: Clarivate Analytics<br><br>Date of Search: 14 January 2022<br><br>Number of hits: 1,606	Field labels<br><br>• TS = title, abstract, author keywords and Keywords Plus<br>• TI = title<br>• SO = source title, journal title<br>• NEAR/x = within x words, regardless of order<br>• * = truncation of word for alternate endings<br><br>Note: sometimes “quotation marks” are needed for single search terms to avoid automatic term mapping (lemmatization).

#	Query	Hits
#1	lupus (Publication Titles)	5,838
#2	TS=(lupus NEAR/2 (neuropsych* or neuro-psych*))	1,371
#3	TS=(SLE AND lupus)	30,206
#4	#1 OR #2 OR #3	33,337
#5	SO=(biomarker* OR marker*)	4,170
#6	TS=(biomarker* or "biological marker*" or "diagnostic marker*")	446,816
#7	TS=((immunol* or metabol* or molecular* or novel) NEAR/2 marker*)	78,147
#8	TS=((blood or cerebrospinal or plasma or sera or serologic* or serum or urinary or urine) NEAR/2 marker*)	39,903
#9	TS=((antibod* or antigen* or autoantibod* or cytokine* or glycoprotein* or immunoglob* or lectin* or lipoprotein* or peptide* or protein* or RNA) NEAR/2 marker*)	33,772
#10	#5 OR #6 OR #7 OR #8 OR #9	574,758
#11	TI=(animal or animals or canine or dog or dogs or feline or hamster or hamsters or lamb or lambs or mice or monkey or monkeys or mouse or murine or pig or pigs or piglet* or porcine or primate or primates or rabbit or rabbits or rat or rats or rodent* or sheep or sheeps or veterinar*)	2,908,411
#12	SO=(animal or animals or canine or dog or dogs or feline or hamster or hamsters or lamb or lambs or mice or monkey or monkeys or mouse or murine or pig or pigs or piglet* or porcine or primate or primates or rabbit or rabbits or rat or rats or rodent* or sheep or sheeps or veterinar*)	171,258
#13	#11 OR #12	3,024,307
#14	TI=(case NEAR/0 (report* or series or stud*))	456,360
#15	#13 OR #14	3,476,405
#16	(#4 AND #10) NOT #15	2,162
#17	(#4 AND #10) NOT #15	1,606
