# Supplementary material for: Biomarkers in Neuropsychiatric Systemic Lupus Erythematosus: A Systematic Literature Review of the Last Decade
Source: Brain Sci. 2022 Jan 30;12(2):192. doi: 10.3390/brainsci12020192 (PMC8869794; doi:10.3390/brainsci12020192)
Supplement: Supplementary file 1 [file brainsci-12-00192-s001.zip › Table S3.pdf]

Table S3. Risk of bias assessment of cross-sectional studies*

Author, year	Were the criteria for inclusion in the sample clearly defined?	Were the study subjects and the setting described in detail?	Was the exposure measured in a valid and reliable way?	Were objective, standard criteria used for measurement of the condition?	Were confounding factors identified?	Were strategies to deal with confounding factors stated?	Were the outcomes measured in a valid and reliable way?	Was appropriate statistical analysis used?
Aibara et al., 2018 (1)	Yes	No	Not applicable†	Yes	No	No	Not applicable	Yes
Bassiouni et al., 2021 (2)	Yes	Yes	Not applicable†	Yes	Yes	Yes	Not applicable	Yes
Engel et al., 2021 (3)	Yes	Yes	Not applicable†	No	No	No	Not applicable	Yes
Fredi et al., 2015 (4)	Yes	No	Not applicable†	No	No	No	Not applicable	Yes
Govoni et al., 2012 (5)	Yes	Yes	Not applicable†	Yes	Yes	Yes	Not applicable	Yes
Hawro et al., 2015 (6)	Yes	No	Not applicable†	Yes	Yes	Yes	Not applicable	Yes
Hawro et al., 2015 (7)	Yes	No	Not applicable†	Yes	Yes	Yes	Not applicable	Yes
Hopia et al., 2011 (8)	Yes	No	Not applicable†	Yes	No	No	Not applicable	Yes
Huang et al., 2020 (9)	Yes	Yes	Not applicable†	Yes	Yes	Yes	Not applicable	Yes
Ichinose et al., 2015 (10)	Yes	Yes	Not applicable†	Yes	No	No	Not applicable	Yes
Ichinose et al., 2018 (11)	Yes	Yes	Not applicable†	Yes	No	No	Not applicable	Yes
Karassa et al., 2000 (12)	Yes	Yes	Not applicable†	Yes	Yes	Yes	Not applicable	Yes
Lu et al., 2021 (13)	Yes	No	Not applicable†	No	Yes	Yes	Not applicable	Yes
Mike et al., 2019 (14)	Yes	No	Not applicable†	No	No	No	Not applicable	Yes
Namjou et al., 2011 (15)	Yes	No	Not applicable†	Yes	Yes	Yes	Not applicable	Yes
Noris-García et al., 2018 (16)	Yes	Yes	Not applicable†	Yes	No	No	Not applicable	Yes
Sharaf-Eldin et al., 2017 (17)	Yes	No	Not applicable†	No	No	No	Not applicable	Yes
Sharaf-Eldin et al., 2020 (18)	Yes	No	Not applicable†	No	Yes	Yes	Not applicable	Yes
Sun et al., 2019 (19)	Yes	Yes	Not applicable†	Yes	Yes	Yes	Not applicable	Yes
Tsuchiya et al., 2014 (20)	Yes	Yes	Not applicable†	Yes	Yes	Yes	Not applicable	Yes

Ushigusa et al., 2016 (21)	Yes	Yes	Not applicable†	Yes	No	No	Not applicable	Yes
van der Meulen et al., 2017 (22)	Yes	No	Not applicable†	Yes	No	No	Not applicable	Yes
Vanarsa et al., (in print) (23)	Yes	No	Not applicable†	No	No	No	Not applicable	Yes

* Assessed by Joanna Briggs Institute (JBI) Critical Appraisal Checklist for Analytical Cross Sectional Studies (24).

† Due to the lack of a gold standard.

References

1. Aibara N, Ichinose K, Baba M, Nakajima H, Satoh K, Atarashi R, Kishikawa N, Nishida N, Kawakami A, Kuroda N, et al. Proteomic approach to profiling immune complex antigens in cerebrospinal fluid samples from patients with central nervous system autoimmune diseases. Clin Chim Acta. 2018;484:26-31.
2. Bassiouni SA, Abdeen HM, Morsi HK, Zaki ME, Abdelsalam M, Gharbia OM. Programmed death 1 (PD-1) serum level and gene expression in recent onset systemic lupus erythematosus patients. The Egyptian Rheumatologist. 2021;43(3):213-8.
3. Engel S, Boedecker S, Marczynski P, Bittner S, Steffen F, Weinmann A, Schwarting A, Zipp F, Weinmann-Menke J, Luessi F. Association of serum neurofilament light chain levels and neuropsychiatric manifestations in systemic lupus erythematosus. Ther Adv Neurol Disord. 2021;14:17562864211051497.
4. Fredi M, Bianchi M, Andreoli L, Greco G, Olivieri I, Orcesi S, Fazzi E, Cereda C, Tincani A. Typing TREX1 gene in patients with systemic lupus erythematosus. Reumatismo. 2015;67(1):1-7.
5. Govoni M, Bombardieri S, Bortoluzzi A, Caniatti L, Casu C, Conti F, De Vita S, Doria A, Farina I, Ferraccioli G, et al. Factors and comorbidities associated with first neuropsychiatric event in systemic lupus erythematosus: does a risk profile exist? A large multicentre retrospective cross-sectional study on 959 Italian patients. Rheumatology (Oxford). 2012;51(1):157-68.
6. Hawro T, Bogucki A, Krupińska-Kun M, Maurer M, Woźniacka A. Serum neuron specific enolase - a novel indicator for neuropsychiatric systemic lupus erythematosus? Lupus. 2015;24(14):1492-7.
7. Hawro T, Bogucki A, Krupińska-Kun M, Maurer M, Woźniacka A. Intractable headaches, ischemic stroke, and seizures are linked to the presence of anti-β2GPI antibodies in patients with systemic lupus erythematosus. PLoS One. 2015;10(3):e0119911.
8. Hopia L, Thangarajh M, Khademi M, Laveskog A, Wallstrom E, Svenungsson E, Andersson M. Cerebrospinal fluid levels of a proliferation-inducing ligand (APRIL) are increased in patients with neuropsychiatric systemic lupus erythematosus. Scand J Rheumatol. 2011;40(5):363-72.
9. Huang Q, Shen S, Qu H, Huang Y, Wu D, Jiang H, Yuan C. Expression of HMGB1 and TLR4 in neuropsychiatric systemic lupus erythematosus patients with seizure disorders. Ann Transl Med. 2020;8(1):9.
10. Ichinose K, Arima K, Ushigusa T, Nishino A, Nakashima Y, Suzuki T, Horai Y, Nakajima H, Kawashiri SY, Iwamoto N, et al. Distinguishing the cerebrospinal fluid cytokine profile in neuropsychiatric systemic lupus erythematosus from other autoimmune neurological diseases. Clin Immunol. 2015;157(2):114-20.
11. Ichinose K, Ohyama K, Furukawa K, Higuchi O, Mukaino A, Satoh K, Nakane S, Shimizu T, Umeda M, Fukui S, et al. Novel anti-suprabasin antibodies may contribute to the pathogenesis of neuropsychiatric systemic lupus erythematosus. Clin Immunol. 2018;193:123-30.

12. Karassa FB, Ioannidis JP, Touloumi G, Boki KA, Moutsopoulos HM. Risk factors for central nervous system involvement in systemic lupus erythematosus. Qjm. 2000;93(3):169-74.
13. Lu L, Kong W, Zhou K, Chen J, Hou Y, Dou H, Liang J. Association of lipoproteins and thyroid hormones with cognitive dysfunction in patients with systemic lupus erythematosus. BMC Rheumatol. 2021;5(1):18.
14. Mike EV, Makinde HM, Gulinello M, Vanarsa K, Herlitz L, Gadhvi G, Winter DR, Mohan C, Hanly JG, Mok CC, et al. Lipocalin-2 is a pathogenic determinant and biomarker of neuropsychiatric lupus. J Autoimmun. 2019;96:59-73.
15. Namjou B, Kothari PH, Kelly JA, Glenn SB, Ojwang JO, Adler A, Alarcón-Riquelme ME, Gallant CJ, Boackle SA, Criswell LA, et al. Evaluation of the TREX1 gene in a large multi-ancestral lupus cohort. Genes Immun. 2011;12(4):270-9.
16. Noris-García E, Arce S, Nardin P, Lanigan ME, Acuña V, Gutierrez F, Robinson-Agramonte MA, Gonçalves CA. Peripheral levels of brain-derived neurotrophic factor and S100B in neuropsychiatric systemic lupus erythematous. Lupus. 2018;27(13):2041-9.
17. Sharaf-Eldin WE, Kishk NA, Gad YZ, Hassan H, Ali MAM, Zaki MS, Mohamed MR, Essawi ML. Extracellular miR-145, miR-223 and miR-326 expression signature allow for differential diagnosis of immune-mediated neuroinflammatory diseases. J Neurol Sci. 2017;383:188-98.
18. Sharaf-Eldin W, Kishk N, Sakr B, El-Hariri H, Refeat M, ElBagoury N, Essawi M. Potential Value of miR-23a for Discriminating Neuromyelitis Optica Spectrum Disorder from Multiple Sclerosis. Arch Iran Med. 2020;23(10):678-87.
19. Sun J, Li X, Zhou H, Liu X, Jia J, Xie Q, Peng S, Sun X, Wang Q, Yi L. Anti-GAPDH Autoantibody Is Associated with Increased Disease Activity and Intracranial Pressure in Systemic Lupus Erythematosus. J. 2019;2019:7430780.
20. Tsuchiya H, Haga S, Takahashi Y, Kano T, Ishizaka Y, Mimori A. Identification of novel autoantibodies to GABA(B) receptors in patients with neuropsychiatric systemic lupus erythematosus. Rheumatology (Oxford). 2014;53(7):1219-28.
21. Ushigusa T, Ichinose K, Sato S, Michitsuji T, Shimizu T, Umeda M, Fukui S, Nishino A, Nakashima Y, Koga T, et al. Soluble α-klotho is a potential biomarker associated with neuropsychiatric systemic lupus erythematosus. Clin Immunol. 2016;165:29-34.
22. van der Meulen PM, Barendregt AM, Cuadrado E, Magro-Checa C, Steup-Beekman GM, Schonenberg-Meinema D, Van den Berg JM, Li QZ, Baars PA, Wouters D, et al. Protein array autoantibody profiles to determine diagnostic markers for neuropsychiatric systemic lupus erythematosus. Rheumatology (Oxford). 2017;56(8):1407-16.
23. Vanarsa K, Sasidharan P, Duran V, Gokaraju S, Nidhi M, Titus ASCLS, Soomro S, Putterman C, Greenberg B, Mok CC, et al. Comprehensive aptamer-based screen of cerebrospinal fluid from Neuropsychiatric Lupus reveals potential biomarkers that overlap with the choroid plexus transcriptome. Arthritis rheumatol. (in print).
24. JBI Critical Appraisal Tools. Available online: https://jbi.global/critical-appraisal-tools (accessed on 19 January 2021).
