# Supplementary material for: Biomarkers in Neuropsychiatric Systemic Lupus Erythematosus: A Systematic Literature Review of the Last Decade
Source: Brain Sci. 2022 Jan 30;12(2):192. doi: 10.3390/brainsci12020192 (PMC8869794; doi:10.3390/brainsci12020192)
Supplement: Supplementary file 1 [file brainsci-12-00192-s001.zip › Table S4.pdf]

Table S4. Risk of bias assessment of meta-analyses*

Author, year	Is the review question clearly and explicitly stated?	Were the inclusion criteria appropriate for the review question?	Was the search strategy appropriate?	Were the sources and resources used to search for studies adequate?	Were the criteria for appraising studies appropriate?	Was critical appraisal conducted by two or more reviewers independently?	Were there methods to minimize errors in data extraction?	Were the methods used to combine studies appropriate?	Was the likelihood of publication bias assessed?	Were recommendations for policy and/or practice supported by the reported data?	Were the specific directives for new research appropriate?
Choi et al., 2020 (1)	Yes	Yes	Yes	Yes	Yes	Yes	Yes	Yes	Yes	Yes	Yes

* Assessed by Joanna Briggs Institute (JBI) Critical Appraisal Checklist for Systematic Reviews and Research Syntheses (2).

References

1. Choi MY, FitzPatrick RD, Buhler K, Mahler M, Fritzler MJ. A review and meta-analysis of anti-ribosomal P autoantibodies in systemic lupus erythematosus. Autoimmun Rev. 2020;19(3):102463.
2. JBI Critical Appraisal Tools. Available online: https://jbi.global/critical-appraisal-tools (accessed on 19 January 2021).
