# Supplementary material for: Biomarkers in Neuropsychiatric Systemic Lupus Erythematosus: A Systematic Literature Review of the Last Decade
Source: Brain Sci. 2022 Jan 30;12(2):192. doi: 10.3390/brainsci12020192 (PMC8869794; doi:10.3390/brainsci12020192)
Supplement: Supplementary file 1 [file brainsci-12-00192-s001.zip › Table S5.pdf]

Table S5. Risk of bias assessment of cohort studies*

Author, year	Were the two groups similar and recruited from the same population?	Were the exposures measured similarly to assign people to both exposed and unexposed groups?	Was the exposure measured in a valid and reliable way?	Were confounding factors identified?	Were strategies to deal with confounding factors stated?	Were the groups/participants free of the outcome at the start of the study (or at the moment of exposure)?	Were the outcomes measured in a valid and reliable way?	Was the follow up time reported and sufficient to be long enough for outcomes to occur?	Was follow up complete, and if not, were the reasons to loss to follow up described and explored?	Were strategies to address incomplete follow up utilized?	Was appropriate statistical analysis used?
Fragoso-Loyo et al, 2012 (1)	Yes	Not applicable	Not applicable †	No	No	Yes	Not applicable	Yes	Yes	Not applicable	Yes
Huang et al., 2016 (2)	Yes	Not applicable	Not applicable †	Yes	Yes	Yes	Not applicable	Yes	Unclear	Unclear	Yes
Ichinose et al., 2016 (3)	Yes	Not applicable	Not applicable †	No	No	Yes	Not applicable	Yes	Yes	Not applicable	Yes
Kitagori et al., 2019 (4)	Not applicable	Not applicable	Not applicable †	No	No	No	Not applicable	No	Yes	No	Yes
Mikdashi et al., 2005 (5)	Yes	Not applicable	Not applicable †	Yes	Yes	Yes	Not applicable	Yes	Unclear	Unclear	Yes
Mok et al., 2001 (6)	Yes	Not applicable	Not applicable †	Yes	Yes	Unclear	Not applicable	Yes	Unclear	Unclear	Yes
Ramirez et al., 2015 (7)	Yes	Not applicable	Not applicable †	No	No	No	Not applicable	Yes	Unclear	Unclear	Yes
Ramirez et al., 201 (8)	Yes	Not applicable	Not applicable †	Yes	Yes	No	Not applicable	Yes	Unclear	Unclear	Yes
Zhang et al., 2021 (9)	Yes	Not applicable	Not applicable †	Yes	Yes	No	Not applicable	Yes	Yes	No	Yes

* Assessed by Joanna Briggs Institute (JBI) Critical Appraisal Checklist for Cohort Studies (10).

† Due to the lack of a gold standard.

References

1. Fragoso-Loyo H, Atisha-Fregoso Y, Núñez-Alvarez CA, Llorente L, Sánchez-Guerrero J. Utility of interferon-α as a biomarker in central neuropsychiatric involvement in systemic lupus erythematosus. J Rheumatol. 2012;39(3):504-9.
2. Huang X, Magder LS, Petri M. Predictors of Incident Seizure in Systemic Lupus Erythematosus. J Rheumatol. 2016;43(3):565-75.
3. Ichinose K, Arima K, Umeda M, Fukui S, Nishino A, Nakashima Y, Suzuki T, Horai Y, Koga T, Kawashiri SY, et al. Predictors of clinical outcomes in patients with neuropsychiatric systemic lupus erythematosus. Cytokine. 2016;79:31-7.
4. Kitagori K, Yoshifuji H, Oku T, Ayaki T, Kuzuya A, Nakajima T, Akizuki S, Nakashima R, Murakami K, Ohmura K, et al. Utility of osteopontin in cerebrospinal fluid as a diagnostic marker for neuropsychiatric systemic lupus erythematosus. Lupus. 2019;28(3):414-22.
5. Mikdashi J, Krumholz A, Handwerger B. Factors at diagnosis predict subsequent occurrence of seizures in systemic lupus erythematosus. Neurology. 2005;64(12):2102-7.
6. Mok CC, Lau CS, Wong RW. Neuropsychiatric manifestations and their clinical associations in southern Chinese patients with systemic lupus erythematosus. J Rheumatol. 2001;28(4):766-71.
7. Ramirez GA, Lanzani C, Bozzolo EP, Citterio L, Zagato L, Casamassima N, Canti V, Sabbadini MG, Rovere-Querini P, Manunta P, et al. TRPC6 gene variants and neuropsychiatric lupus. J Neuroimmunol. 2015;288:21-4.
8. Ramirez GA, Coletto LA, Bozzolo EP, Citterio L, Delli Carpini S, Zagato L, Rovere-Querini P, Lanzani C, Manunta P, Manfredi AA, et al. The TRPC6 intronic polymorphism, associated with the risk of neurological disorders in systemic lupus erythematous, influences immune cell function. J Neuroimmunol. 2018;325:43-53.
9. Zhang S, Li M, Zhang L, Wang Z, Wang Q, You H, Wang Y, Li M, Zeng X. Clinical Features and Outcomes of Neuropsychiatric Systemic Lupus Erythematosus in China. J. 2021;2021:1349042.
10. JBI Critical Appraisal Tools. Available online: https://jbi.global/critical-appraisal-tools (accessed on 19 January 2021).
